# Effectiveness of High Fidelity Video-Assisted Real-Time Simulation: A Comparison of Three Training Methods for Acute Pediatric Emergencies

**DOI:** 10.1155/2012/709569

**Published:** 2012-02-22

**Authors:** Ester H. A. J. Coolen, Jos M. T. Draaisma, Marije Hogeveen, Tim A. J. Antonius, Charlotte M. L. Lommen, Jan L. Loeffen

**Affiliations:** ^1^Department of Pediatric Surgery, Radboud University Nijmegen Medical Centre, 6500 HB Nijmegen, The Netherlands; ^2^Department of Pediatrics, Radboud University Nijmegen Medical Centre, 6500 HB Nijmegen, The Netherlands

## Abstract

*Background*. Video-assisted real-time simulation (VARS) offers the possibility of developing competence in acute medicine in a realistic and safe environment. We investigated the effectiveness of the VARS model and compared it with educational methods like Problem-Based Learning (PBL) and Pediatric Advanced Life Support (PALS). *Methods*. 45 fourth-year medical students were randomized for three educational methods. Level of knowledge and self-efficacy were measured before and after intervention. Clinical performance was measured by a blinded observer using a video checklist of prescripted scenarios on a high-fidelity simulator. *Results*. Knowledge test and self-efficacy scores improved significantly (*P* < 0.001) without differences between educational groups. The VARS group showed significantly (*P* < 0.05) higher scores on both postintervention scenarios concerning structure and time. *Conclusion*. VARS training is an effective educational method teaching pediatric acute care skills in the undergraduate curriculum. When compared to PBL and PALS training, VARS training appears to be superior in enhancing short-term clinical performance.

## 1. Background

The introduction of high-fidelity patient simulation into medical education programs for residents and medical staff has occurred at a rapid pace in training programs of disciplines with complex patient environments like anesthesiology, pediatrics, and emergency medicine [1–7]. Simulation provides a learner-focused and safe educational environment, without patient risk. It enables us to design clinical training experiences tailored to medical discipline and level of experience. Therefore, patient simulation is considered to be an excellent educational tool to prepare junior doctors for daily clinical practice [8–11]. However, evidence on the effectiveness of high-fidelity simulation training in the undergraduate curriculum is scarce. One randomized controlled trial compared high-fidelity simulation to problem-based learning (PBL) [12]. The authors concluded that simulation-based training was superior to PBL for the acquisition of critical assessment and management skills. In other studies, which compared simulation-based training to educational models as traditional lecturing, screen-based simulation and video-assisted learning, high-fidelity simulation was not superior [13–15]. One study investigated the comparative effectiveness of different simulation-based acute care training methods and found no significant advantage of using whole-body manikins over computer screen-based training and simple part-task manikins. However, full-scale patient simulation appeared to be more effective in transferring previously learned clinical skills into new emergency situations [16]. These mixed results can probably best be explained by different levels of experience in participants and variability in context and learning goals of educational programs. This emphasizes the importance of selecting the right time and use for this type of sophisticated and relatively expensive educational tool during the medical curriculum.

All previously mentioned trials focused on the management of critically ill adult patients. These results cannot easily be extrapolated to pediatric medicine, considering the differences between adult and pediatric critical care management. Pediatric resuscitation primarily relies on early recognition and treatment of respiratory rather than circulatory events, which requires a different focus. Also inpatient resuscitation in children is uncommon, providing few opportunities to bring acquired skills into practice [17, 18]. To overcome the paucity of exposure to real-emergency situations and promote retention of skills, structural practice in a simulated learning environment with immediate feedback should take place repeatedly [19].

Video-assisted real-time simulation (VARS) offers the possibility of developing competence in acute pediatric medicine without relying on clinical exposure alone. In a realistic and safe environment, students have to recognize and manage an acute pediatric problem and perform their skills in real time on a high-fidelity simulator. This is followed by a structured video-assisted debriefing, during which feedback is given. The VARS concept is well suited to train for emergency situations which are rare, but for which a certain level of preparedness and self-confidence is essential.

We designed a study to evaluate the effectiveness of a single VARS training in pediatric emergencies in undergraduate medical students. In this prospective, blinded, and randomized trial, we compared high-fidelity VARS training to other more traditional educational models (problem-based learning (PBL) and scenario training) using an advanced life support (ALS) manikin as used in the European Pediatric Life support Course (EPLS) provider for skills training in acute pediatric care.

## 2. Methods

### 2.1. Study Design, Setting, and Population

**Figure 1:**
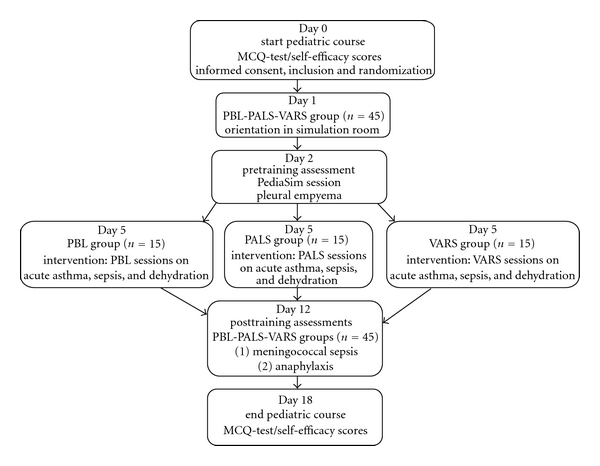
Overview of study design.

 The concept of our medical curriculum is based on national normative goals concerning learning outcomes according to the competency-based CanMEDS framework. The skills necessary for the recognition and treatment of the seriously ill infant, child, and adult are trained in the Skill and Simulation Unit of our centre during the Master's degree program (fourth- and sixth-year students). This study was conducted in the Skills and Simulation Unit of our centre. Scenarios took place using a high-fidelity patient simulator (PediaSIM by METI) in a simulation theater equipped with cameras and microphones. We used a scripted nurse in all three scenarios. A total of 88 fourth-year medical students enrolled in a three-week preclerkship general pediatric course using problem-based learning. During this course one day was spent to familiarize students with the assessment and management of children with acute life-threatening problems. A total of 63 students gave written informed consent to participate in our trial. Our study was limited to 45 students, which were randomly selected to enroll in our study. They were informed that performance assessments generated from the study would not affect their course evaluation. The study was approved by the Radboud University Medical Faculty Board. Students were blindly randomized in three different educational groups (group 1: PBL, group 2: PALS, and group 3: VARS). Students not included in our trail received training according to PBL principles without participating in simulated scenarios. Absence during any portion of the training resulted in exclusion from the study. An overview of the study design is shown in Figure 1.

### 2.2. Orientation Phase

 After randomization, students participated in a standardized introduction session in the simulation room reviewing the features of the simulator and available equipment. They were given the opportunity to familiarize with the simulator and simulation room, the use of equipment, and practice skills (e.g., auscultation and iv access).

### 2.3. Pretraining Assessment

The pretraining assessment took place after the orientation phase in our simulation room. The setting of the pretraining scenario consisted of an acute respiratory problem (pleural empyema). Students were expected to individually recognize and manage altered vital signs as dyspnea, tachycardia, and desaturation on a high fidelity simulator (PediaSIM by METI). The content of this scenario was based on predesigned curricular objectives and had a maximal duration of 10 minutes. After the pretraining assessment, students received no video debriefing or feedback. Performance of students was recorded on video for offline video scoring by blinded raters (medical staff) on acute care skills using a standardized checklist.

### 2.4. Educational Intervention

 Following the orientation phase and pretraining assessment, all students received an interactive introduction lecture on acute pediatric medicine. After this lecture group 1 (*n* = 15) engaged in self-directed learning based on a written case stem including three patient cases in acute pediatric medicine (acute asthma, sepsis, and dehydration). Group 2 (*n* = 15) practiced scenarios on an advanced life support pediatric manikin (Megacode kid by Laerdal) on the same topics as listed in group 1. The “low fidelity” features of the manikin caused the need for receiving oral information from an instructor present in the room during the scenario. Afterwards these students received feedback on their performance by an instructor based on recalling the participant's actions afterwards. Group 3 (*n* = 15) trained on a high-fidelity patient simulator (PediaSIM) using computer-based scenarios on the same three topics as listed in group 1. The responses to the student's actions were displayed on the patient monitor in a real-time fashion. These training sessions were videotaped and discussed by an instructor during a debriefing. All groups received the same amount of educational time (5 hours) in their educational environment. All instructors were experienced pediatricians and educators (PALS and VARS instructor) and used the same, previously established, learning objectives. Following the intervention session, all students received an interactive closing lecture. The learning objectives of acute asthma, sepsis, and dehydration were discussed and evaluated with the opportunity to ask questions.

### 2.5. Posttraining Assessment 

On day 12 of the pre-clerkship course all included students (*n* = 45) participated in two final assessment scenarios. The format of the post-training assessments was identical to the pretraining assessment where again each student had to manage a critically ill child in a 10-minute prescripted scenario. The first posttraining scenario was a meningococcal sepsis and had a similar content to one of the three central study themes (sepsis). The second assessment scenario, anaphylactic shock, presented a new problem for all students and was designed to study the development of problem-solving skills, as neither group had explicitly practiced the management of this condition during the educational intervention. However, in the introduction lecture, all students received information about the recognition and management of anaphylactic shock. During all scenarios, group members were separated in order to avoid sharing of information about scenario content. We chose not to give any feedback on student performance directly after post-training assessments in order to avoid influence of the educational effect of the assessment.

### 2.6. Knowledge and Self-Efficacy Tests

At the start and end of the pre-clerkship pediatric course, all students were tested on their knowledge of acute pediatric management by a 45-item MCQ test designed by the Dutch Foundation for the Emergency Medical care for Children, range from 0 to 100%. Also every student filled out a self-efficacy form on their pediatric resuscitation skills before and after the pediatric course. We used a validated Visual Analogue Scale (VAS), range from 0 to 100 mm, to measure self-efficacy at the start and end of the pediatric course [20].

### 2.7. Checklist Scoring 

A standardized checklist unique for every scenario consisted of items on an ABC-structured approach, duration of primary survey, therapy, reassessment, and diagnosis. To generate a weighted score-higher-point values were assigned to critical actions (Table 2). Critical actions are actions that have a direct effect on outcome and survival of the patient when not performed in time (based on European Resuscitation Council (ERC) guidelines for Advanced Pediatric Life Support 2005). All checklists items where scored in a yes or no manner by a rater not otherwise involved in the students' curriculum and blinded to group assignment. A total score per scenario was calculated for each student (percentage of checklist items performed) with a range from 0 to 100%. To test the reliability of our checklists, the pretraining and both post-training scenarios of 30 randomly selected students were scored by a second blinded rater. Students and instructors did not have access to the checklist forms until the study was completed. 

### 2.8. Statistical Analysis 

Descriptive statistics were calculated for students' knowledge and self-efficacy. We used repeated measures ANOVA to investigate a significant change in MCQ and VAS scores before and after the pediatric course. To compare clinical performance between educational groups, a total checklist score per scenario was calculated for each student. A repeated measures ANOVA was used to investigate significant changes in pre- and post training assessments and significant differences in checklist scores between educational groups (PBL, PALS, VARS). We chose a repeated measures ANOVA, because all students participated three times in a simulated scenario. Using a standard ANOVA to compare means would not be appropriate in this case because it fails to model the correlation between the repeated measures (the ANOVA assumption of independence would be violated). A *P*-value <0.05 was considered significant for all statistical tests. An Intra Class Correlation (ICC) coefficient for each scenario was computed to test the interrater agreement and test the reliability of our checklists in detecting students' growth in clinical performance. Data were analyzed using SPSS 16.0.

## 3. Results

A total of 45 students voluntarily enrolled in our study during a three-month period. Two students were not able to attend the intervention session and were excluded from our study, leaving 43 students for evaluation. 

**Figure 2:**
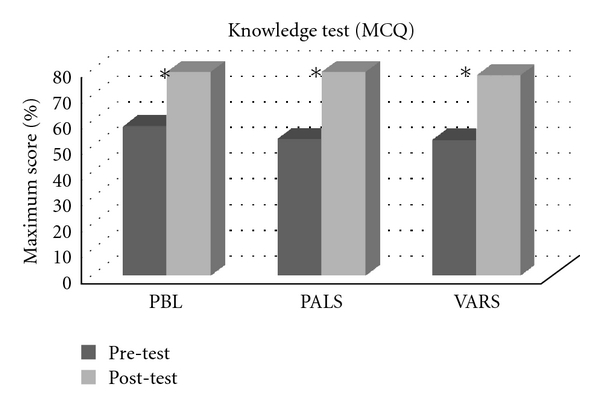
Change in MCQ scores (from 0 to 100% of maximum score) before and after the pediatric training program significant increase (*P* value < 0.001). No significant differences between groups (*P*  value = 0.48).

**Figure 3:**
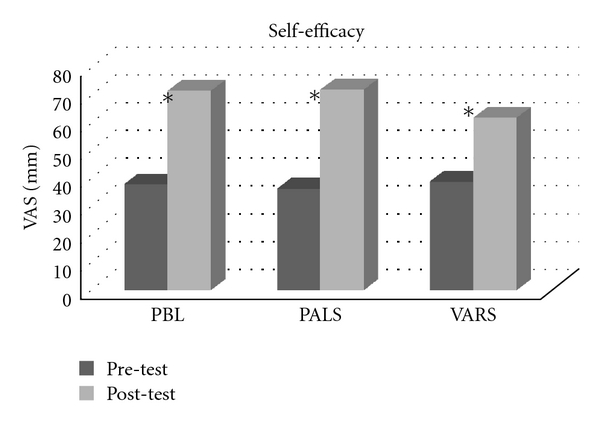
Change in self-efficacy VAS scores (from 0 to 100 mm visual analogue scale) before and after the pediatric training program significant increase (*P* value < 0.001). No significant differences between groups (*P* value = 0.40).

Knowledge test (MCQ) scores improved significantly (*P* < 0.001) following intervention without significant differences between the three study groups (*P* = 0.48) as shown in Figure 2. There was also an overall improvement in self-efficacy scores after intervention (*P* < 0.001), again without significant differences between groups (*P* = 0.40) (Figure 3). Although we found no statistical differences between educational groups, Figure 3 shows a slightly less increase in self-efficacy scores in the VARS group compared to the other groups.

The checklist scores on clinical performance in both groups indicated no basis for rejecting a normal distribution. The mean pretraining clinical assessment scores were comparable between the three study groups. The mean scores of the post-training assessment scenarios increased significantly compared to the pretraining assessments for all study groups (*P* < 0.001) (Table 1).

**Figure 4:**
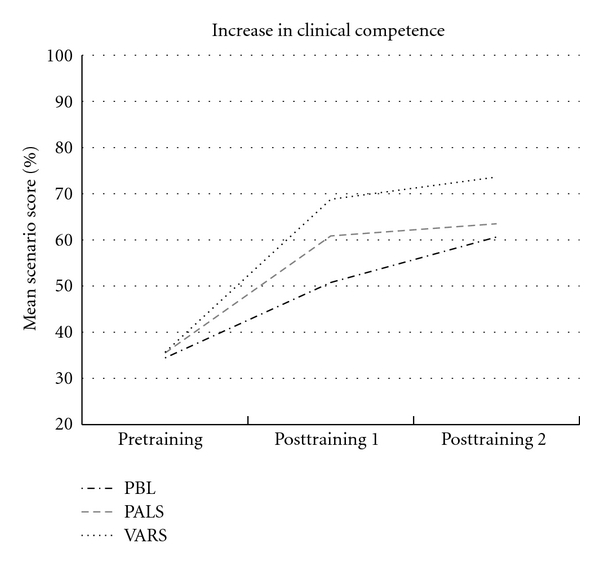
Increase in clinical performance (means, percentage of maximum score) from the preintervention scenario (1) to both postintervention scenarios. These results show significant differences between educational group mean scores (*P* < 0.001). Also learning curves per educational group are statistically different (*P* < 0.01).

We found significant differences between educational groups (*P* < 0.05). Increase in clinical performance from the preintervention scenario to both postintervention scenarios is shown in Figure 4. In this figure the VARS group shows the steepest learning curve, with significantly the best improvement on checklist scores (*P* < 0.05).

The percentage of interrater agreement on checklist scores was excellent with an Intraclass Correlation Coefficient of 0.95 on scenario 1 (pleural empyema), 0.94 on scenario 2 (meningococcal sepsis), and 0.91 on scenario 3 (anaphylaxis) after randomly scoring 66% of all scenarios by a second rater using a weighted yes/no checklist.

## 4. Discussion

Students were eager to commit themselves to the study and made many positive comments about the opportunity to practice clinical skills in a safe environment. They reported that the training sessions with specific and direct feedback on clinical skills and problem management (PALS and VARS) motivated them to improve themselves and increased their confidence before entering a clinical clerkship. According to literature, this is a very important observation because improved self-efficacy encourages positive thinking, allowing a person to visualize successful performance and is likely to increase a doctor's motivation to continuously improve resuscitation competence [21]. 

However, we also demonstrated improved knowledge and self-efficacy on acute pediatric skills after a one-day training session, regardless of the educational model that was used. One could even say that self-efficacy in acute management of children tends to increase less after high-fidelity VARS training compared to the increase seen following problem-based learning and PALS training although we could not establish a statistical significant difference. Possibly this effect can be explained by the fact that during VARS training students are more exposed to the stress of actually having to perform their actions real time and quickly respond to the changing status of the patient. This could help students to recognize how much they still do not know after a single training event and how difficult it can be to adhere to a structured approach during stressful circumstances. This observation is supported by previous research indicating that participation in simulated emergency situations does increase the perceived need of junior doctors for more supervision and assistance of other team members [22].

Our main results show improved skill acquisition in students trained on a high-fidelity simulator using the VARS method compared to PBL or PALS training. The primary differences in the VARS group were the realistic real-time environment and the opportunity to give individual feedback on clinical performance based on the video-taped scenario. The actual equipment, a Pediasim manikin and monitors which modeled physiological changes in vital parameters, provided a very authentic situation for the student. A facilitating instructor, always present in the room during the PALS scenarios, wasn't necessary during the VARS training, and feedback and physiological changes were obtained from the patient and monitor during the scenario. This likely evokes a more engaged approach to the patient's problem by the students participating in the VARS training. During video debriefing formative feedback is given, while the students can watch their performance on a screen underlined by changes in the condition of the patient and his vital parameters. Individualized feedback with video-taped material of actual actions is a very important aspect of learning clinical reasoning and skills [23]. Ideally, students and residents would receive regular formative feedback on their performance from their supervisors, but, in day-to-day clinical practice, this is not easily accomplished [24, 25]. High workload and the necessity to act promptly in emergency situations have an unfavorable effect on the ability of supervisors to engage in debriefing or individualized feedback. The structured approach of VARS training can both assist in identification of training needs and provide training for the intervention with feedback and an individualized learning path as a very powerful tool to improve clinical competence.

At this point, our single intervention without long-term followup, seriously limits conclusions about retention of skills. To maintain and enhance skills, students need repeat exposure to simulation. Repeated training and participant followup until finishing their pediatric clerkship could have filtered out the training effect of our assessments and answered questions about long-term outcomes.

 Another limitation is the fact that all assessments were conducted in a simulated setting and not during actual patient care. Considering the low frequency of pediatric emergencies, the use of highly realistic simulations is considered to be the best alternative up to this point. Of course the high-fidelity patient simulator has face validity [26, 27], but there are concerns that performance of junior doctors in a simulated environment, were there are fewer cues to correct performance, may result in a worse performance than might otherwise take place. On the other hand, it could be argued that the simulated environment, without the stress of dealing with a living patient that might die, may result in an enhanced performance. To ultimately study whether high-fidelity simulation training actually makes a difference to patient outcome and whether it will be cost effective will be a challenge. Nonetheless, these are important questions to be answered. Internationally medical university institutions are on a tight budget, and funding a high-fidelity simulator, with well-trained instructors for ongoing refresher training sessions for medical personnel, is expensive. 

However, supervisors of care should ask themselves whether health care workers, who cannot demonstrate a structured and safe performance on a high-fidelity simulator, should be allowed to perform these skills on real patients. That's why these costs should be considered in the light of a shared investment in patient safety and quality of care. 

## 5. Conclusions

This study of acquisition of skills in managing an acutely ill child using three different teaching methods indicates that video-assisted real-time simulation is an effective educational tool in the undergraduate curriculum. All training methods resulted in improved knowledge, self-efficacy, and clinical skills, but VARS training significantly improved clinical performance compared to PBL and PALS training in the short term. Long-term effects should be measured during a followup study.

## Figures and Tables

**Table 1:** Clinical performance scores by educational group.

Group	PBL (*n* = 14)	PALS (*n* = 14)	VARS (*n* = 15)	*P* value
Pretraining	34,43 (11,65)	35,50 (7,99)	35,67 (13,36)	0.953
Posttraining 1	50,79 (9,50)	60,86 (9,00)	68,80 (9,63)	0.000
Posttraining 2	60,64 (13,4)	63,50 (13,20)	73,60 (11,34)	0.013

Pretraining scores (means and standard deviations) were equivalent for the PBL, PALS, and VARS group (*P* value = 0.95). There is a significant increase in mean checklist scores before and after intervention for all educational groups (*P* value < 0.001). Also these results show a significant difference between educational groups with VARS group showing the highest checklist scores on both posttraining assessment 1 and 2 (*P*  value resp. <0.001 and 0.01).

**Table 2:** Example checklist scenario 2 (meningococcal sepsis). Scoring takes place in a yes or no manner with an item score ranging from 0 to 2.— areas indicate critical actions. These critical item scores are tripled, creating a weighted score. A total score per scenario was calculated for each student (percentage of checklist items performed) with a range from 0 to 100%.

	Airway management	Score

(1) Assess airway	0-no assessment 2-look/listen/feel, talks to pt	

(2) Oxygen applied (high-flow NRM)	0- no oxygen applied 1->60 seconds 2-<60 seconds	—

	Breathing	

(1) Asks for signs of respiratory distress	0-no 1-yes	

(2) Assess respiratory rate	0-no 1-yes	

(3) Auscultation	0-no 1-yes	

(4) Assess thorax excursions	0-no 1-yes	

(5) Percussion	0-no 1-yes	

(6) Checks monitor for saturation	0-no 1-yes	

	Circulation	

(1) Checks monitor for BP and HR (time to recognition of vitals)	0-no monitoring 1->60 seconds 2-<60 seconds	

(2) Asses pulses (central/distal)	0-no 1-incomplete 2-yes	

(3) Assess CRT (5 seconds sternum)	0-no 1-incorrect 2-correct	

(4) Time to IV access	0-no access 1->5 minutes 2-<5 minutes	—

*Duration primary survey (ABC completed)*	0->5 minutes 1-1–5 minutes 2-<1 minute	—

*Adherence to ABC structure*	0-no 1-partially 2-yes	—

	Therapy	

(1) Antibiotic therapy	0-no or incorrect dose 1-yes, asks pediatrician for dose 2-yes, and knows correct dose	—

(2) Dexamethasone	0-no 1-yes, asks pediatrician for dose 2-yes, and knows correct dose	

(3) Blood culture	0-no 1-yes	

(4) Checks glucose	0-no 2-yes	

(5) Fluid bolus (20 mL/kg NaCl 0.9%)	0-no 1->5 minutes 2-< 5 minutes	—
	Therapy	

(6) Recognizes need for second fluid bolus	0-no 2- yes	

(7) Proposes inotrope therapy after fluid resuscitation	0-no 2-yes	

*Reassess ABC*	0-no 2-yes	

*Diagnosis (meningococcal sepsis)*	0-incorrect 2-correct	—

	Total score	
	% maximum (67)	

## References

[1] Good ML (2003). Patient simulation for training basic and advanced clinical skills. *Medical Education*.

[2] Boulet JR, Murray D, Kras J, Woodhouse J, McAllister J, Ziv A (2003). Reliability and validity of a simulation-based acute care skills assessment for medical students and residents. *Anesthesiology*.

[3] Ziv A, Ben-David S, Ziv M (2005). Simulation based medical education: an opportunity to learn from errors. *Medical Teacher*.

[4] Langhan TS, Rigby IJ, Walker IW, Howes D, Donnon T, Lord JA (2009). Simulation-based training in critical resuscitation procedures improves residents’ competence. *Canadian Journal of Emergency Medicine*.

[5] Morgan PJ, Cleave-Hogg D (2005). Simulation technology in training students, residents and faculty. *Current Opinion in Anaesthesiology*.

[6] Fiedor ML (2004). Pediatric simulation: a valuable tool for pediatric medical education. *Critical Care Medicine*.

[7] Falcone RA, Daugherty M, Schweer L, Patterson M, Brown RL, Garcia VF (2008). Multidisciplinary pediatric trauma team training using high-fidelity trauma simulation. *Journal of Pediatric Surgery*.

[8] Chopra V, Gesink BJ, de Jong J, Bovill JG, Spierdijk J, Brand R (1994). Does training on an anaesthesia simulator lead to improvement in performance?. *British Journal of Anaesthesia*.

[9] Fraser K, Peets A, Walker I (2009). The effect of simulator training on clinical skills acquisition, retention and transfer. *Medical Education*.

[10] Issenberg SB, Scalese RJ (2008). Simulation in health care education. *Perspectives in Biology and Medicine*.

[11] Murray DJ, Boulet JR, Kras JF, Woodhouse JA, Cox T, McAllister JD (2004). Acute care skills in anesthesia practice: a simulation-based resident performance assessment. *Anesthesiology*.

[12] Steadman RH, Coates WC, Yue MH (2006). Simulation-based training is superior to problem-based learning for the acquisition of critical assessment and management skills. *Critical Care Medicine*.

[13] Nyssen AS, Larbuisson R, Janssens M, Pendeville P, Mayné A (2002). A comparison of the training value of two types of anesthesia simulators: computer screen-based and mannequin-based simulators. *Anesthesia and Analgesia*.

[14] Morgan PJ, Cleave-Hogg D, McIlroy J, Devitt JH (2002). Simulation technology: a comparison of experiential and visual learning for undergraduate medical students. *Anesthesiology*.

[15] Gordon JA, Shaffer DW, Raemer DB, Pawlowski J, Hurford WE, Cooper JB (2006). A randomized controlled trial of simulation-based teaching versus traditional instruction in medicine: a pilot study among clinical medical students. *Advances in Health Sciences Education*.

[16] Owen H, Mugford B, Follows V, Plummer JL (2006). Comparison of three simulation-based training methods for management of medical emergencies. *Resuscitation*.

[17] Grant EC, Marczinski CA, Menon K (2007). Using pediatric advanced life support in pediatric residency training: does the curriculum need resuscitation?. *Pediatric Critical Care Medicine*.

[18] Trainor JL, Krug SE (2000). The training of pediatric residents in the care of acutely ill and injured children. *Archives of Pediatrics and Adolescent Medicine*.

[19] Ericsson KA (2008). Deliberate practice and acquisition of expert performance: a general overview. *Academic Emergency Medicine*.

[20] Turner NM, van de Leemput AJ, Draaisma JMT, Oosterveld P, Ten Cate OTJ (2008). Validity of the visual analogue scale as an instrument to measure self-efficacy in resuscitation skills. *Medical Education*.

[21] Maibach EW, Schieber RA, Carroll MFB (1996). Self-efficacy in pediatric resuscitation: implications for education and performance. *Pediatrics*.

[22] Marteau TM, Wynne G, Kaye W, Evans TR (1990). Resuscitation: experience without feedback increases confidence but not skill. *British Medical Journal*.

[23] Srinivasan M, Hauer KE, Der-Martirosian C, Wilkes M, Gesundheit N (2007). Does feedback matter? Practice-based learning for medical students after a multi-institutional clinical performance examination. *Medical Education*.

[24] Remmen R, Denekens J, Scherpbier A (2000). An evaluation study of the didactic quality of clerkships. *Medical Education*.

[25] Yarris LM, Linden JA, Hern HG (2009). Attending and resident satisfaction with feedback in the emergency department. *Academic Emergency Medicine*.

[26] Issenberg SB, McGaghie WC, Petrusa ER, Gordon DL, Scalese RJ (2005). Features and uses of high-fidelity medical simulations that lead to effective learning: a BEME systematic review. *Medical Teacher*.

[27] Morgan PJ, Cleave-Hogg D, DeSousa S, Tarshis J (2004). High-fidelity patient simulation: validation of performance checklists. *British Journal of Anaesthesia*.

